# HBV reactivation during immunotherapy for hepatocellular carcinoma: risk factors and clinical management

**DOI:** 10.3389/fimmu.2026.1765054

**Published:** 2026-02-10

**Authors:** Yurou Jin, Chao Jin, Ronghui Xie, Jingming Zhang, Guangmin Wei, Ling Zheng

**Affiliations:** 1 Department of Infectious Diseases, Mengchao Hepatobiliary Hospital of Fujian Medical University, Fuzhou, China; 2 Department of Hepatology and Infectious Diseases, Mengchao Hepatobiliary Hospital of Fujian Medical University, Fuzhou, China; 3 The Second Clinical Medical College, Nanchang University, Nanchang, China; 4 Department of Infectious Diseases, Fuzhou Second General Hospital, Fuzhou, China; 5 Department of Oncology, Mengchao Hepatobiliary Hospital of Fujian Medical University, Fuzhou, China

**Keywords:** clinical management, HBV reactivation, hepatocellular carcinoma, immunotherapy, risk factor

## Abstract

Hepatitis B virus reactivation (HBVr) poses a serious clinical challenge and potentially life-threatening complication in patients with hepatocellular carcinoma (HCC), particularly amid the expanding use of modern immunotherapeutic agents. Despite progress in antiviral prophylaxis and refined risk-stratification strategies, HBVr continues to compromise treatment efficacy and survival outcomes, especially in patients receiving immune checkpoint inhibitors, tyrosine kinase inhibitors, or combination regimens. This review comprehensively synthesizes current evidence on the virological foundations, clinical risk factors, and immunopathological mechanisms underpinning HBVr during HCC treatment, emphasizing the pivotal roles of covalently closed circular DNA (cccDNA) persistence and treatment-induced immune dysregulation. We further examine the comprehensive evidence of risk factors in HBVr, including various treatments for HCC. We also reviewed the clinical consequences of HBVr, including acute hepatocellular injury, unplanned treatment discontinuations, and adverse long-term HCC prognosis. Evidence-based management approaches, such as universal serological screening, individualized antiviral prophylaxis, and multidisciplinary coordination, are detailed to effectively reduce reactivation risk. Finally, we discuss emerging therapeutic strategies, including HBV-specific cellular therapies and innovative siRNA-based and immunostimulatory cytokine delivery platforms, which offer promising avenues for eradicating viral reservoirs and restoring immune surveillance.

## 1 Introduction

Hepatocellular carcinoma (HCC) continues to pose a heavy global health burden, ranking as the sixth most diagnosed cancer and the third leading cause of cancer-related mortality worldwide (1). A principal driver of this burden is the persistent endemicity of hepatitis B virus (HBV) infection, which constitutes a major etiological factor in hepatocarcinogenesis. As of 2022, approximately 254 million individuals worldwide were estimated to be living with chronic HBV infection (2). China bears a disproportionately high share of this burden, accounting for nearly one-third of global chronic HBV infections, despite a substantial decline in prevalence, from historical peaks to 5.86% in 2020, attributable to comprehensive vaccination programmed (3). Nevertheless, the global incidence of HCC continues to rise, with approximately 870,000 new cases reported in 2022; modelling studies project a potential doubling of cases to 1.52 million by 2050 in the absence of intensified interventions (4). HBV infection alone drives nearly 39% of all HCC cases globally, underscoring its dominant role in the disease’s etiology (4). Mortality associated with viral hepatitis remains staggering, resulting in an estimated 1.3 million deaths annually, with HBV-associated liver diseases accounting for 1.1 million deaths in 2022 (5, 6).

While the epidemiological burden is well-established, a deeper mechanistic understanding of HBV-related hepatocarcinogenesis remains essential. Chronic HBV infection promotes oncogenesis through multiple synergistic pathways. HBV DNA integration into the host genome functions as a potent insertional mutagen, disrupting critical regulatory genes and driving chromosomal instability (7). Concurrently, viral proteins, notably HBx and truncated pre-S/S variants, directly perturb cellular processes by modulating proliferation, apoptosis, and DNA repair mechanisms (8). Furthermore, the chronic inflammatory microenvironment induced by persistent viral replication accelerates hepatocyte turnover and promotes fibrotic progression, thereby establishing a permissive niche for malignant transformation (9, 10). As a non-cytopathic virus, HBV primarily mediates hepatic injury through immune-driven mechanisms. It impairs both innate and adaptive immune responses, exacerbating chronic inflammation and facilitating carcinogenesis; clinical observations indicate that patients with significant intrahepatic inflammatory activity following HBV infection face a substantially elevated risk of developing HCC (11).

The complex interplay between viral persistence and immune dysregulation sets the stage for a serious clinical complication in HCC management: hepatitis B virus reactivation (HBVr). HBVr is defined as an abrupt loss of immunological control over chronic or resolved HBV infection, leading to viral rebound and hepatitis flare (12). This phenomenon typically arises when immunosuppression, triggered by anticancer therapies, immunomodulatory agents, or the malignancy itself, disrupts immune surveillance, culminating in sharply elevated HBV DNA levels and frequently marked increases in serum alanine aminotransferase (ALT). In cases of previously resolved infection, HBVr may involve reverse seroconversion, characterized by reappearance of HBsAg, and can precipitate severe acute hepatitis, hepatic decompensation, or even fulminant liver failure (13). The clinical implications of HBVr are profound (14), often necessitating the interruption or discontinuation of potentially curative treatments, including surgical resection, transplantation, systemic agents, and locoregional therapies, which compromises treatment outcomes (15). Hepatitis flares associated with HBVr contribute significantly to morbidity and mortality, particularly in patients with underlying cirrhosis, further complicating clinical management (16). Notably, numerous anticancer therapies, including immune checkpoint inhibitors, tyrosine kinase inhibitors, cytokine inhibitors, CAR-T-cell immunotherapies, and corticosteroids, have been associated with an elevated risk of HBVr in HBsAg-positive patients (17).

Urgently, advancements in HCC treatment, particularly in immunotherapy, have introduced new dimensions of risk. The expanding use of immunotherapeutic agents, especially immune checkpoint inhibitors (ICIs) and adoptive cell therapies, has become a cornerstone of advanced HCC management (18). However, their mechanisms of action, often involving T-cell reactivation and reversal of exhaustion, may inadvertently disrupt immune control of HBV. Emerging clinical evidence indicates a tangible risk of HBVr during ICIs treatment, even among patients with occult or resolved infections (19). This presents a critical dilemma: how can clinicians maximize oncological efficacy while mitigating the life-threatening risk of HBVr (20). In addressing this urgent clinical need, our review will discuss the biological mechanisms underpinning HBVr during immunotherapy and its interplay with HCC development, summarize current epidemiological trends and risk factors of HBVr in HCC, and explore the impacts of HBVr on HCC immunotherapy. Furthermore, we will propose evidence-based framework for monitoring and prophylaxis, and outline future directions for both research and clinical practice.

A literature search was conducted in PubMed and Web of Science for articles published up to October 2025. Search terms included “hepatocellular carcinoma,” “HBV reactivation,” “immunotherapy,” “immune checkpoint inhibitors,” “targeted therapy,” “TACE,” “hepatectomy,” and their combinations. We included clinical studies (prospective or retrospective) that involved HBV−related HCC patients receiving various anticancer treatments (surgery, locoregional therapy, chemotherapy, targeted therapy, immunotherapy, etc.) and reported the incidence of HBV reactivation. Case reports, animal studies, non−English publications, and articles whose full text was unavailable were excluded. If multiple publications reported on the same patient cohort, the study with the largest sample size or longest follow−up was selected to avoid duplication.

## 2 Biology of HBVr and its interplay with HCC development

**Figure 1:**
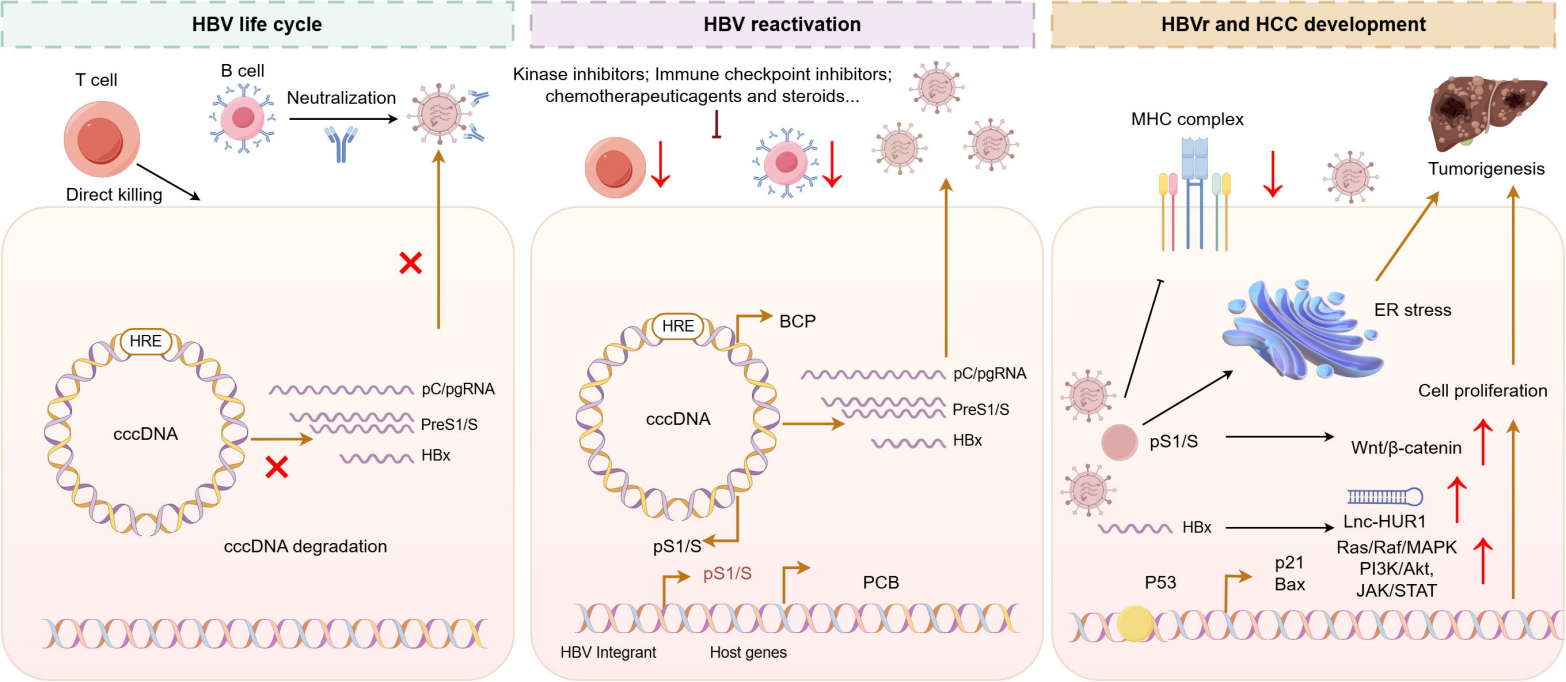
Molecular mechanisms of HBV persistence, reactivation triggers, and crosstalk with HCC pathogenesis. The persistence of HBV relies on the robust stability and transcriptional activity of cccDNA, a mini-chromosome within infected hepatocytes that evades current antiviral therapies. Viral reactivation can be triggered by immunosuppressive or immunomodulatory agents, including cytotoxic chemotherapy, that impair immune surveillance through depletion of B or T lymphocytes. This breakdown in viral control promotes hepatocarcinogenesis via multiple mechanisms: the HBx protein dysregulates key signaling pathways (e.g., PI3K/AKT, MAPK, Wnt/β-catenin, and JAK/STAT) and compromises DNA repair; PreS/S mutant proteins induce endoplasmic reticulum stress and genomic instability; and viral integration disrupts host genome integrity through insertional mutagenesis, promoting the HCC development.

The remarkable persistence of HBV is fundamentally underpinned by the exceptional stability of covalently closed circular DNA (cccDNA), a chromatin-like episome that resides within the nucleus of infected hepatocytes (21). Serving as the primary transcriptional template for all viral RNAs, cccDNA is organized into a minichromosome through the association with histones and viral proteins, thereby acquiring structural and functional characteristics that confer resilience against host innate immune recognition and current antiviral agents (22). This persistence mechanism allows cccDNA to evade immunological clearance and resist therapeutic elimination, establishing a long-lasting reservoir that predisposes individuals to viral reactivation following immunosuppression (23). The maintenance of viral latency relies critically on sustained and multispecific host immune responses (24). Virus-specific CD8^+^ T cells play a central role by directly targeting infected hepatocytes and secreting antiviral cytokines, while neutralizing antibodies against hepatitis B surface antigen (HBsAg) contribute to the containment of viral spread (24). These coordinated immune effectors exert continuous pressure to suppress viral replication and gene expression, effectively holding the virus in a state of functional inactivity (25). The precarious balance between viral persistence and host immunologic control represents a defining feature of HBV pathogenesis, dictating the course of infection and the risk of reactivation upon disruption of immune surveillance (26) (**Figure 1**).

HBVr represents a significant clinical complication that arises when iatrogenic or malignancy-associated immunosuppression disrupts the delicate equilibrium sustaining viral latency under host immune surveillance (27). This breakdown in immunological control enables viral rebound and is commonly precipitated by a diverse array of immunomodulatory agents. Broad-spectrum immunosuppressive drugs, along with increasingly utilized targeted anticancer therapies, can profoundly undermine antiviral defenses through mechanisms such as direct lymphocyte depletion, suppression of cytokine-mediated signaling pathways, and the induction of functional T cell exhaustion (28). Corticosteroids, for instance, exert dual effects: they transiently repress viral transcription via interaction with glucocorticoid-responsive elements embedded within the HBV genome, while concurrently impairing T-cell effector functions, thereby creating a permissive environment for unreserved viral replication (29). Similarly, therapies that target B cells, such as anti-CD20 monoclonal antibodies, significantly diminish the production of neutralizing antibodies against HBV envelope proteins, leading to a decline in humoral immunity and impaired neutralization of circulating viral particles (30, 31). Notably, immune checkpoint inhibitors, designed to reinvigorate antitumor immunity by blocking inhibitory receptors such as PD-1 or CTLA-4, may inadvertently disrupt the precise immunoregulatory balance that maintains HBV containment (32, 33). By reversing T-cell exhaustion in an antigen-nonspecific manner, these agents can precipitate an abrupt and severe loss of virological control, even among patients with previously resolved or occult HBV infection, highlighting the multifaceted vulnerability of the HBV-immune interface to therapeutic perturbation (34). HBVr often precipitates significant liver injury, clinically observed as a hepatitis flare (35). According to the prevailing model, this hepatocellular damage stems not from direct viral cytopathic effects, but rather from a rapid immune reconstitution following the withdrawal of immunosuppression, a process analogous to immune reconstitution inflammatory syndrome (36). As the immune system recovers, reinvigorated virus-specific T cells mount a vigorous response against infected hepatocytes exhibiting high viral antigen loads, resulting in extensive apoptotic and necroinflammatory cell death (37). This process is further amplified by innate immune activation and cytokine-driven inflammation, which exacerbate lobular injury and may culminate in acute hepatic decompensation, particularly in individuals with pre-existing cirrhosis (38). The relationship between HBVr and HCC is multifaceted, involving both direct viral oncogenic mechanisms and indirect inflammation-associated pathways (39). HBV contributes to carcinogenesis through integrated viral DNA that disrupts key host tumor suppressor genes and oncogenes, as well as through the actions of viral proteins such as HBx and mutated preS/S, which perturb normal cellular functions including proliferation, apoptosis, and DNA repair (40). Simultaneously, the chronic inflammatory microenvironment, characterized by repeated cycles of immune-mediated hepatocyte destruction and regenerative proliferation, establishes a pro-oncogenic milieu (41). Reactivation events may further accelerate HCC progression by inducing surges of inflammatory activity, enhancing local immunosuppression, and fostering the clonal expansion of pre-malignant hepatocytes (42). Growing clinical evidence underscores that HBVr is associated with increased rates of HCC recurrence and worse overall survival, emphasizing the imperative of sustained viral suppression in the comprehensive management of HBV-related HCC (43).

## 3 Clinical epidemiology and risk factors of HBVr in HCC

Building upon the immunovirological mechanisms described above, the following section outlines the clinical risk factors associated with HBVr in patients undergoing specific HCC treatments. HBVr during anti-cancer therapy represents a significant clinical challenge that markedly exacerbates hepatic morbidity and mortality in HCC patients. The epidemiological landscape of HBVr in this vulnerable population demonstrates considerable variability, with documented incidence rates ranging from 4% to 67% among chronic carriers undergoing conventional chemotherapy (19). This remarkably wide range reflects the complex interplay of multiple factors, including the heterogeneity of treatment regimens, varying baseline host characteristics, and differences in monitoring protocols across clinical studies. Beyond conventional chemotherapy, emerging evidence indicates that HBVr has been increasingly identified in diverse clinical settings including hepatectomy, local ablation therapies, and novel systemic therapeutic approaches (44). The probability of reactivation exhibits a direct correlation with the degree of immunosuppression induced by anticancer treatments and is further modulated by intricate interactions between treatment-related factors, viral characteristics, and individual patient attributes (45, 46). The clinical significance of HBVr extends beyond the viral replication itself to encompass substantial clinical consequences (47). A considerable proportion of patients experiencing reactivation develop significant liver dysfunction during critical periods of anti-cancer therapy, frequently necessitating interruption or modification of potentially life-prolonging treatments (48). Such treatment disruptions not only compromise oncological outcomes but may also lead to severe hepatitis, acute liver failure, and increased mortality rates, particularly in patients with pre-existing liver cirrhosis (49). The risk of HBVr is governed by a sophisticated multidimensional framework incorporating host, tumor, and treatment-related determinants, further complicated by the ongoing absence of universally standardized prophylactic protocols across clinical settings (50, 51).

### 3.1 Hepatectomy and local ablation therapy or ethanol injection as risk factors

In the context of HCC treatment, both hepatectomy and local ablation therapies, including radiofrequency ablation (RFA) and ethanol injection, are significant risk factors for HBVr, as evidenced by numerous clinical studies (**Table 1**). Hepatectomy, particularly partial or anatomical liver resection, is associated with a considerable risk of HBVr, with reported rates varying widely across studies due to differences in patient populations, surgical extent, follow-up duration, and definitions of reactivation. For instance, reactivation rates range from as low as 2.9% in a cohort of 34 patients (52) to 40.8% in a study of 130 patients (53), though most large-scale studies report rates between 14% and 21.5% (54, 55). Notably, a study involving 1,609 patients reported a reactivation rate of 19.1% (56), while another with 857 patients noted 21.5% (55), underscoring the substantial risk posed by surgical resection. The follow-up periods in these studies vary from 1 to 53 months, with most reactivation events occurring within the first year post-surgery. The diagnostic criteria for HBVr also differ, with most studies defining it as either a >1 log increase in HBV DNA levels or reappearance of HBV DNA in previously undetectable patients, often with a threshold of >200 IU/mL based on previous guidelines. Some studies specifically require these changes to occur within 1 to 3 months post-operatively, highlighting the temporal proximity of reactivation to surgical stress and immune modulation. Besides, local ablation therapies, such as radiofrequency ablation and percutaneous ethanol injection, carry a lower but still notable risk of HBVr (**Table 1**). The reported rates generally range from 0% to 11.6%, with one study of 43 patients reporting 11.6% reactivation after local ablation therapy over a median follow-up of 11 months (57). Radiofrequency ablation, in particular, has been associated with reactivation rates of 5.6% in a study of 125 patients and 10.8% in a larger cohort of 538 patients followed for nearly 46 months (54, 58). Interestingly, ethanol injection appears to pose a minimal risk, with one study of 9 patients reporting no reactivation events, though the small sample size limits generalizability (52). The definition of reactivation in these studies is consistent with that used in surgical cohorts, typically involving a significant rise in HBV DNA or viral reappearance. The relatively lower risk compared to hepatectomy may be attributed to the less invasive nature of ablation techniques, which cause more localized tissue damage and potentially induce less systemic immunosuppression or inflammatory response. However, the risk remains clinically relevant, particularly in patients with high baseline viral load or inadequate antiviral prophylaxis.

**Table 1:** Summary of hepatectomy and local ablation therapy as risk factors for HBVr in patients with HCC.

Treatment type	Treatment details	Study design (R/P)	No of patients	HBVr rate	Follow-up (months)	Testing standard of HBVr	Ref.
Hepatectomy	Liver resection	R/P	25	7/25 (28%)	36	HBV DNA ≥ 5-fold increase within 3 months	(96)
	Liver resection	R	34	1/34 (2.9%)	13	HBV DNA > 1 log increase or reappearance	(52)
	Liver resection	R	77 (82 resections)	7/82 (8.5%)	20	Detectable HBV DNA without other causes	(97)
	Partial hepatectomy	R	1609	308/1609 (19.1%)	12	HBV DNA > 1 log increase or reappearance (>200 IU/mL)	(56)
	Liver resection	R	101	18/101 (17.8%)	3	HBV DNA > 2 log increase within 3 months	(98)
	Liver resection	R	130	53/130 (40.8%)	28	HBV DNA > 1 log increase or reappearance	(53)
	Partial hepatectomy	P	164	10/164 (6.1%)	1	NA	(99)
	Liver resection	P	84	15/84 (31.8%)	NA	NA	(100)
	Liver resection	R	93	13/93 (14%)	NA	HBV DNA > 1 log increase or reappearance	(54)
	Liver resection	R	204	19/204 (9.3%)	NA	HBV DNA > 1 log increase or reappearance (>200 IU/mL)	(65)
	Liver resection	R	112	6/112 (5.4%)	6	NA	(101)
	Liver resection	P	135	26/135 (19.3%)	1	HBV DNA > 1 log increase or reappearance	(102)
	Liver resection	R	258	50/258 (19.4%)	1	HBV DNA > 1 log increase or reappearance	(103)
	Liver resection	R	174	32/174 (18.4%)	1	HBV DNA reappearance	(104)
	Liver resection	P/R	574	87/574 (15.2%)	53	HBV DNA > 1 log increase or reappearance (>200 IU/mL) within 3 months	(105)
	Liver resection	R	209	31/209 (14.8%)	1	HBV DNA > 1 log increase or reappearance within 1 month	(106)
	Liver resection	R	538	99/538 (18.4%)	NA	HBV DNA > 1 log increase or reappearance (>200 IU/mL)	(107)
	Liver resection	R	857	184/857 (21.5%)	NA	NA	(55)
Local ablation therapy or ethanol injection	Local ablation therapy	P	43	5/43 (11.6%)	11	HBV DNA > 1 log increase	(57)
	Radiofrequency ablation	R	125	7/125 (5.6%)	NA	HBV DNA > 1 log increase or reappearance	(54)
	Ethanol injection	R	9	0/9 (0%)	13	HBV DNA > 1 log increase or reappearance	(52)
	Percutaneous radiofrequency ablation	R	538	58/538 (10.8%)	45.9	HBV DNA ≥ 10-fold increase within 3 months	(58)

Overall, both hepatectomy and local ablation therapies are established risk factors for HBVr in HCC patients, with hepatectomy conferring a higher risk likely due to greater parenchymal loss, surgical stress, and associated immunosuppression (59, 60).

### 3.2 Chemotherapy and radiotherapy as risk factors

Transarterial chemoembolization (TACE), hepatic arterial infusion chemotherapy (HAIC), and radiotherapy are also significant iatrogenic risk factors for HBVr in patients undergoing treatment for HCC, with varying rates reported across numerous studies due to differences in treatment protocols, patient characteristics, and definitions of reactivation (**Table 2**). Among chemotherapy-based interventions, TACE is the most extensively studied modality, with reactivation rates demonstrating considerable variability, ranging from 4.3% in a prospective study of 69 patients to as high as 38.9% in a recent retrospective analysis of 108 patients (61, 62). Larger cohort studies generally report intermediate rates; for instance, a study of 1,547 patients observed a reactivation rate of 9.2% over a median follow-up of 16.5 months (63), whereas other sizable cohorts of 162, 183, and 386 patients reported rates of 35.2%, 26.2%, and 14.8%, respectively (52, 64, 65). This wide range can be attributed to several factors, including the chemotherapeutic agents used, the intensity and number of TACE sessions, baseline viral load, and liver function status. The definition of reactivation is highly consistent across these studies, typically characterized by a greater than 1 log10 increase in HBV DNA levels or the reappearance of detectable viremia, often with a specific threshold such as >200 IU/mL. Notably, HAIC, another locoregional chemotherapy approach, appears to confer a high risk, with one retrospective study of 106 patients reporting a reactivation rate of 30.2% within just three months of treatment, underscoring the potent stimulatory effect of cytotoxic chemotherapy on viral replication in the context of immunosuppression (66).

**Table 2:** Summary of chemotherapy and radiotherapy as risk factors for HBVr in patients with HCC.

Treatment type	Treatment details	Study design (R/P)	No of patients	HBVr rate	Follow-up (months)	Testing standard of HBVr	Ref.
Chemotherapy	TACE	R	33	8/33 (24%)	1	NA	(108)
	TACE	P	69	3/69 (4.3%)	2	HBV DNA reappearance	(62)
	TACE	P	73	16/73 (21.9%)	5.8	HBV DNA > 1 log increase	(109)
	TACE	P	162	57/162 (35.2%)	11	HBV DNA > 1 log increase	(57)
	TACE	R	83	28/83 (33.7%)	13	HBV DNA > 1 log increase or reappearance	(52)
	TACE	R	228	33/228 (14.5%)	1.7	HBV DNA > 1 log increase or reappearance (>200 IU/mL)	(110)
	TACE	P	183	48/183 (26.2%)	31	NA	(64)
	TACE	R	386	57/386 (14.8%)	3	HBV DNA > 1 log increase or reappearance (>200 IU/mL)	(65)
	TACE	P	109	23/109 (21.1%)	NA	HBV DNA > 1 log increase or reappearance	(111)
	TACE	R	1547	143/1547 (9.2%)	16.5	HBV DNA > 1 log increase	(63)
	TACE	P	170	25/170 (14.7%)	3	HBV DNA > 1 log increase or reappearance (>200 IU/mL)	(112)
	TACE	R	108	42/108 (38.9%)	1	HBV DNA > 1 log increase	(61)
	HAIC	R	106	32/106 (30.2%)	3	HBV DNA > 1 log increase or reappearance	(66)
Radiotherapy	RT	P	133	17/133 (12.7%)	2.3	HBV DNA > 1 log increase or reappearance (>200 IU/mL)	(67)
	3D-CRT	R	48	7/48 (14.6%)	2	HBV DNA > 2 log increase	(68)
	RT	R	90	20/90 (22.2%)	3.7	HBV DNA > 1 log increase or reappearance	(113)
	CRT	R	69	17/69 (24.6%)	3.7	HBV DNA > 1 log increase or reappearance	(114)

3D-CRT, three dimension- conformal radiotherapy; TACE, transarterial chemoembolization; HAIC, hepatic arterial infusion chemotherapy; RT, radiotherapy; NA, not available; P, prospective; R, retrospective.

Radiotherapy, including three-dimensional conformal radiotherapy (3D-CRT), conventional radiotherapy (RT), and conformal radiotherapy (CRT), also poses a definite, though potentially modest, risk for HBVr in HCC patients (**Table 2**). The reported reactivation rates range from 12.7% to 24.6%, as observed in studies with sample sizes between 48 and 133 patients (67, 68). The mechanisms are thought to involve radiation-induced liver injury and a localized inflammatory response that may disrupt immune control of the virus. The follow-up periods in these studies are generally shorter than those for TACE, often spanning only a few months post-treatment, which may suggest a more immediate onset of reactivation following radiation exposure. The diagnostic criteria align with those used for chemotherapy, predominantly relying on a significant rise in HBV DNA or viral reappearance.

In summary, both locoregional chemotherapy (TACE and HAIC) and radiotherapy are established significant risk factors for HBVr in HCC patients. The risk associated with chemotherapy, particularly TACE, shows considerable heterogeneity but is substantiated by numerous large studies, whereas the risk from radiotherapy is consistently reported at an intermediate level. These findings highlight the critical necessity of stringent virological monitoring and the mandatory implementation of prophylactic antiviral therapy for all HBV-infected HCC patients undergoing these treatments to mitigate the risk of reactivation and its associated hepatic complications.

### 3.3 Targeted therapy and immunotherapy as risk factors

The emergence of targeted therapy and immunotherapy has revolutionized the treatment landscape for HCC, yet their roles as potential risk factors for HBVr present a complex and clinically significant challenge (**Table 3**). Among targeted agents, tyrosine kinase inhibitors (TKIs), most notably sorafenib, are associated with a variable but notable risk of HBVr. The reported reactivation rates range widely, from 0% in a smaller cohort of 45 patients to 30% in a larger retrospective study of 296 patients followed for 12 months (69, 70). This substantial variability can be attributed to several factors, including the specific TKIs used, the duration of therapy, underlying patient virology, and crucially, the differing definitions of reactivation employed across studies. Most studies define reactivation as a greater than 1 log10 increase in HBV DNA levels, though some utilize more stringent thresholds, such as a 100-fold increase or reappearance of viremia above 1000 IU/mL, which naturally yields lower incidence rates (71). The larger and more contemporary studies suggest the risk may be more modest; for instance, a study of 419 patients reported a reactivation rate of 12.5% over a prolonged follow-up of 48 months (72). The proposed mechanism for TKIs is not solely rooted in profound immunosuppression but may involve a complex interplay of off-target immunomodulatory effects and direct impact on liver function, potentially disrupting the delicate immune-mediated control of latent HBV infection (47).

**Table 3:** Summary of systemic therapies as risk factor for HBVr in patients with HCC.

Treatment type	Treatment details	Study design (R/P)	No of patients	HBVr rate	Follow-up (months)	Testing standard of HBVr	Ref.
Targeted therapy	TKIs	R	296	89/296 (30%)	12	HBV DNA > 1 log increase	(78)
	TKIs	R	419	52/419 (12.5%)	48	HBV DNA detectable or reappearance (>100 IU/mL)	(72)
	Sorafenib	R	45	0/45 (0%)	3	HBV DNA > 1 log increase	(70)
	Sorafenib	R	130	9/130 (6.9%)	NA	HBV DNA ≥ 1 log increase or HBV DNA > 10^5^ cp/mL	(115)
	Sorafenib	R	78	4/78 (5.1%)	NA	HBV DNA ≥ 2-fold increase	(116)
	Sorafenib	R	156	11/156 (7%)	NA	HBV DNA≥100-fold increase or reappearance (>1000 IU/mL)	(117)
Immunotherapy	Nivolumab or pembrolizumab	P/R	60	4/60 (6.7%)	10.4	HBV DNA > 1 log increase or reappearance (>1000 IU/mL) or HBsAg reappearance	(118)
	Anti-PD-1 therapy	R	120	0/120 (0%)	20	NA	(73)
	Anti-PD-1 therapy	R	202	7/202 (3.5%)	NA	NA	(119)
	Anti-PD-1 therapy	R	218	16/218 (7.4%)	NA	NA	(120)
	Anti-PD-1 or anti-PD-L1 therapy	R	28	1/28 (3.6%)	4.2	HBV DNA ≥ 1 log increase or ≥3–4 logs	(121)
	Immune checkpoint inhibitors	R	62	6/62 (9.7%)	14	HBV DNA > 1 log increase or reappearance or HBsAg reappearance	(74)
	Immune checkpoint inhibitors	R	409	2/409 (0.5%)	68	HBV DNA≥100-fold increase or reappearance (> 1000 IU/mL)	(75)

TKIs, tyrosine kinase inhibitor; PD-1, programmed death 1; PD-L1, programmed death-ligand 1; NA, not available; P, prospective; R, retrospective.

In contrast, immunotherapy, particularly ICIs such as anti-PD-1, anti-PD-L1, nivolumab, and pembrolizumab, appears to carry a comparatively lower and less frequent risk of HBVr, though it remains a critical consideration (**Table 3**). The reactivation rates in published studies are generally low, ranging from 0% to 9.7%, with most large cohorts reporting rates between 3.5% and 7.4% (73, 74). A notable study of 409 patients receiving ICIs reported a very low reactivation rate of only 0.5% over an extensive follow-up period of 68 months (75). This relative infrequency is somewhat paradoxical given the fundamental mechanism of ICIs, which involves reversing T-cell exhaustion and potentially enhancing immune responses against viral antigens (76). It is hypothesized that this very restoration of antiviral immunity might, in some cases, lead to controlled viral clearance without clinical reactivation, or conversely, in rare instances, trigger an immune reconstitution inflammatory syndrome (IRIS)-like event that manifests as reactivation (77). The follow-up durations in these studies are also heterogeneous, and the definition of reactivation occasionally includes the reappearance of HBsAg, providing a broader clinical endpoint.

Overall, while both targeted therapies and immunotherapies are associated with a risk of HBVr in HCC patients with chronic HBV infection, the risk profile and underlying mechanisms differ. TKIs demonstrate a more variable and potentially higher risk, necessitating vigilant monitoring. Immunotherapy, despite its mechanism of action, is associated with a lower observed incidence, though the potential for severe immune-mediated hepatitis underscores the necessity of continued vigilance. These findings affirm the imperative of universal HBV screening, close virological monitoring, and the adherence to prophylactic antiviral therapy throughout the course of these treatments to mitigate the risk of HBVr and its associated morbidity.

### 3.4 Combination therapy as risk factor

The integration of combination therapies, particularly those involving ICIs, TKIs, and locoregional treatments, represents an advanced frontier in HCC management but concurrently introduces a multifaceted risk profile for HBVr. Evidence from recent studies indicates that the risk of HBVr during combination treatment is substantial and varies considerably, with reported rates generally ranging from 10% to 20%, though specific regimens and prophylactic measures can significantly influence outcomes (**Table 4**). For instance, combinations such as TKIs plus anti-PD-1 have been associated with reactivation rates as high as 21.3% in a cohort of 203 patients over 12 months (78), whereas regimens like HAIC combined with lenvatinib and anti-PD-1 showed a lower incidence of 7.5% in 213 patients (79). The triple combination of HAIC, TKIs, and ICIs, even alongside prophylactic entecavir, still resulted in a notable reactivation rate of 14.3% among 307 patients (46), underscoring the challenge of complete risk mitigation despite antiviral prophylaxis. In contrast, some regimens like durvalumab plus tremelimumab demonstrated no reactivation events in a prospective study of 30 patients, though the small sample size necessitates cautious interpretation (80). The definition of reactivation across these studies is often stringent, commonly entailing a significant viral DNA increase (e.g., ≥100-fold or >1 log10 increase) or reappearance above thresholds like 200 or 1000 IU/mL, with follow-up durations varying from 12 to 48 months. The elevated risk associated with many combination strategies likely stems from synergistic immunosuppressive or immunomodulatory effects: TKIs may impair immune surveillance, while ICIs potentially incite immune reconstitution-related flares, and the addition of locoregional therapies like HAIC or TACE can induce further hepatic inflammation and stress. Therefore, although combination therapies markedly improve oncological outcomes in HCC, they concurrently demand intensified virological vigilance and strict adherence to evidence-based antiviral prophylaxis to preempt HBVr-related complications.

**Table 4:** Summary of combination therapy as risk factor for HBVr in patients with HCC.

Treatment type	Treatment details	Study design (R/P)	No of patients	HBVr rate	Follow-up (months)	Testing standard of HBVr	Ref.
Combination therapy	HAIC + lenvatinib + anti-PD1	R	213	16/213 (7.5%)	20	NA	(79)
	HAIC + TKIs + ICIs + entecavir	R	307	44/307 (14.3%)	20	HBV DNA≥10-fold increase or reappearance (>200 IU/mL)	(46)
	TKIs + anti-PD1	R	203	43/203 (21.3%)	12	HBV DNA > 1 log increase	(78)
	Atezolizumab + bevacizumab	R	329	10/329 (2%)	NA	HBV DNA≥100-fold increase or reappearance (>1000 IU/mL)	(117)
	Lenvatinib + camrelizumab	R	216	24/216 (11.1%)	13.8	HBV DNA≥100-fold increase or reappearance (>1000 IU/mL)	(122)
	TACE + TKIs + ICIs	R	119	12/119 (10.1%)	40	NA	(123)
	Durvalumab + tremelimumab	P	30	0/30 (0%)	NA	HBV DNA≥100-fold increase or reappearance (>1000 IU/mL)	(80)
	TKIs + anti-PD1	R	458	76/458 (16.6%)	48	HBV DNA detectable or reappearance (>100 IU/mL)	(4)

TKIs, tyrosine kinase inhibitor; HAIC, hepatic arterial infusion chemotherapy; TACE, Transarterial chemo-embolization; ICIs, immune checkpoint inhibitors; NA, not available; P, prospective; R, retrospective; PD-1, programmed death 1.

In conclusion, HBVr in HCC patients is a preventable yet serious complication influenced by a confluence of treatment, host, and viral factors (20). The wide range of reactivation rates underlines the importance of universal screening, tailored prophylaxis, and continuous monitoring (35). Future studies should standardize definitions and management protocols to harmonize prevention strategies across diverse therapeutic contexts. Ultimately, integrating antiviral stewardship into oncology care is essential to improving outcomes in this vulnerable population. The absence of appropriate antiviral prophylaxis persists as the single most significant modifiable risk factor for HBVr (45). Despite established international guidelines recommending prophylactic NAs for patients undergoing high-risk immunosuppressive therapies, real-world implementation remains inconsistent due to various barriers including insufficient awareness, diagnostic challenges, and resource limitations. Prophylaxis with contemporary agents such as entecavir, tenofovir disoproxil fumarate, or tenofovir alafenamide demonstrates remarkable effectiveness in preventing reactivation, preserving hepatic function, and ensuring uninterrupted delivery of potentially curative anti-cancer therapies (11). Risk stratification instruments like the PAGE-B score, incorporating age, gender, and platelet count, provide valuable frameworks for identifying high-risk patients who would derive maximum benefit from preemptive therapy (81). Emerging serological biomarkers (including AFP and PIVKA-II) and novel assays measuring lgG2 anti-HBc levels (LG2m) promise to further refine risk stratification precision and optimize prophylactic strategies (82, 83).

## 4 Impact of HBVr on HCC immunotherapy

HBVr during immunotherapy for HCC poses a considerable clinical challenge, especially in regions with high endemic rates of HBV infection. Although immunotherapies, particularly ICIs, have transformed the management of advanced HCC, their interplay with HBV can precipitate viral reactivation, potentially undermining both hepatic function and antitumor responses. Clinical evidence confirms that the risk of HBVr during ICIs therapy, though variable, is non-negligible. For example, one study involving 114 HBsAg-positive patients receiving PD-1/PD-L1 inhibitors reported a reactivation rate of 5.3%, with a median time to onset of 18 weeks (84). A broader systematic review of 187 patients with HBV or HCV infection documented grade 3 or 4 transaminase elevations in 3.4% of HBV-positive individuals, although no direct fatal outcomes were linked to HBVr in this cohort. Critically, the lack of prophylactic antiviral therapy emerged as a major risk factor, increasing the odds of reactivation significantly (OR 17.50) (34), underscoring the imperative of initiating and maintaining antiviral prophylaxis throughout immunotherapy.

HBVr exerts a profound impact on outcomes in HCC patients receiving immunotherapy. Reactivation can trigger hepatitis flares, deteriorate liver function, and necessitate interruption of anticancer treatment, thereby compromising disease control. A multicenter study of patients treated with combined HAIC, TKIs, and ICIs observed HBVr in 14.3% of cases despite entecavir prophylaxis (46). Affected patients faced significantly worse survival outcomes, with a median OS of 13.2 months compared to 20.5 months in those without reactivation (46). This adverse prognostic effect was corroborated in a study involving patients with undetectable baseline HBV DNA, in which reactivation correlated with reduced median OS (23 vs. 36 months) and impaired progression-free survival (72). The mechanisms through which HBVr worsens outcomes are multifactorial, encompassing direct virus-induced liver injury, dysregulation of immune homeostasis, and interruptions in effective immunotherapy.

The risk of HBVr is also modulated by the specific immunotherapy regimen employed. Combination strategies, especially those integrating ICIs with TKIs or locoregional modalities, are associated with elevated reactivation rates relative to monotherapies. A retrospective analysis noted reactivation in 21.3% of patients receiving TKIs plus anti-PD-1 therapy, compared to 12.5% under TKIs monotherapy (72). Similarly, another investigation reported 12-month cumulative reactivation rates of 30.0% for combination therapy versus 21.3% for TKIs alone (78), implying that enhanced immunomodulation may potentiate viral replication. Nonetheless, risk levels vary among combination regimens; for instance, a prospective trial of durvalumab and tremelimumab in 30 patients with chronic active hepatitis B documented no reactivation events when combined with entecavir (80), emphasizing the protective efficacy of adequate antiviral prophylaxis.

Effective management of HBVr in the context of HCC immunotherapy necessitates vigilant monitoring and preemptive antiviral strategies. Prophylactic administration of nucleos(t)ide analogues (NAs), such as entecavir or tenofovir, markedly reduces reactivation incidence. A large-scale study in HBsAg-positive patients receiving ICIs demonstrated a reactivation rate of only 0.4% among those receiving antiviral prophylaxis, versus 6.4% in untreated individuals (75). For patients experiencing breakthrough reactivation despite prophylaxis, optimizing antiviral therapy is essential. Evidence suggests that introducing tenofovir alafenamide (TAF) to existing regimens can improve outcomes, with one study reporting an increase in median OS from 8.1 months to 17.9 months following TAF initiation (46). Novel approaches, such as HBV-specific TCR-T cells (e.g., SCG101), also hold promise by targeting both malignant and virus-infected hepatocytes, potentially delivering concurrent antitumor and antiviral activity (85).

In summary, HBVr constitutes a substantial impediment to the success of immunotherapy in HBV-related HCC, detrimentally affecting liver function and overall survival. The risk is especially pronounced with combination immunotherapies, yet it can be substantially mitigated through universal HBV screening, consistent antiviral prophylaxis, and diligent virological monitoring. Future research should prioritize the refinement of antiviral strategies and the development of integrated therapies that simultaneously target HCC and HBV to improve clinical outcomes in this vulnerable population.

## 5 Latest management framework of HBVr of HCC

The contemporary management of HBVr in patients with HCC should follow a risk-adapted framework based on recent international guidelines, including those from the American Gastroenterological Association (AGA) and the European Association for the Study of the Liver (EASL) (46, 86). Clinically, this approach involves performing universal HBV screening before treatment initiation, stratifying patients according to their risk of HBVr, initiating antiviral prophylaxis in high- and moderate-risk individuals, and conducting regular long-term monitoring of HBV DNA and liver function to promptly detect and manage HBVr, thereby reducing treatment-related hepatic complications (87).

A key component of this strategy is universal HBV screening, which should be performed before initiating any immunosuppressive or locoregional therapy for HCC. Recommended serological tests include hepatitis B surface antigen (HBsAg), antibody to hepatitis B core antigen (anti-HBc), and antibody to HBsAg (anti-HBs) to accurately determine a patient’s HBV infection status (13, 46). This screening is critical not only for identifying HBsAg-positive patients but also for detecting HBsAg-negative, anti-HBc-positive individuals who remain at risk for reverse seroconversion. In seropositive patients, further assessment with quantitative HBV DNA testing and liver function tests is essential to establish baseline viral replication and hepatic reserve, enabling informed decisions regarding antiviral prophylaxis and monitoring strategies (31, 88, 89).

Risk stratification serves as the foundation for subsequent clinical decisions. Current guidelines categorize patients into high-, moderate-, and low-risk groups based on the immunosuppressive potential of the planned HCC treatment and baseline serological markers. High-risk regimens, with HBVr rates exceeding 10%, include certain cytotoxic chemotherapies (e.g., anthracycline-based regimens), B-cell-depleting agents such as rituximab, and combined immunotherapy approaches (46). For instance, combinations of TKIs with ICIs have been linked to reactivation rates as high as 21.3%. In such high-risk scenarios, prophylactic antiviral therapy is mandatory. Moderate-risk categories, associated with HBVr rates between 1% and 10%, include monotherapy with ICIs or TACE; here, antiviral prophylaxis is generally recommended, though intensified monitoring may be considered for selected patients, such as those who are HBsAg-negative/anti-HBc-positive without advanced fibrosis (86). Low-risk therapies, such as localized radiotherapy, carry HBVr rates below 1% and typically require only routine monitoring without prophylaxis (46).

For high- and moderate-risk patients, prompt initiation of prophylaxis with a high-barrier-to-resistance NA, such as entecavir, tenofovir disoproxil fumarate (TDF), or tenofovir alafenamide (TAF), is strongly advised per AGA, EASL, and Asian-Pacific Association for the Study of the Liver (APASL) guidelines (13, 46, 86). Prophylaxis should begin before commencing immunosuppressive treatment and continue for a predetermined period after its cessation: at least 6 months for most agents, and extending to 12 months or longer for B-cell-depleting therapies due to their prolonged immunosuppressive effects. Real-world evidence supports the effectiveness of this strategy, demonstrating that NA prophylaxis significantly reduces reactivation rates without substantial renal or metabolic adverse effects (13, 46, 86).

Long-term monitoring remains essential throughout and after HCC treatment. Serial measurement of HBV DNA and alanine aminotransferase (ALT) levels every 3–4 weeks during active immunosuppression, and periodically thereafter, is recommended (20). In HBsAg-positive patients, annual quantitative HBsAg tracking may also be valuable. Such surveillance facilitates early detection of virological breakthrough, prompting timely adjustment of antiviral therapy. Management of breakthrough reactivation entails optimizing antiviral regimens, often by improving adherence or switching to alternative agents, and addressing any associated hepatitis flares (46).

A multidisciplinary collaborative approach involving hepatologists, oncologists, and infectious disease specialists is critical to optimizing patient outcomes. This ensures that HBVr management is effectively integrated into the overall anticancer treatment plan, balancing oncological efficacy with hepatological safety.

In summary, the modern management framework for HBVr in HCC patients advocates a proactive, risk-stratified, and multidisciplinary approach. Universal screening, risk-tailored prophylaxis with high-barrier NAs, appropriate treatment duration, and sustained monitoring are essential to preserve liver function and allow completion of intended cancer therapies. Future research should aim to refine risk categorization, particularly for novel immunotherapeutic agents, and to optimize the long-term cost-effectiveness of prophylactic strategies.

## 6 Future perspectives & conclusions

The available evidence linking HBVr to impaired oncological outcomes, such as reduced overall survival and progression-free survival, stems largely from retrospective observational studies. While these investigations consistently identify an association, they remain susceptible to residual confounding from factors including heterogeneity in liver function, tumor burden, antiviral strategies, and treatment adherence. Furthermore, inconsistencies in the definitions of reactivation, monitoring schedules, and the initiation of antiviral prophylaxis introduce additional variability and potential bias. Thus, although HBV reactivation is a clinically significant event that often accompanies poorer prognosis, current data cannot establish a causal relationship. Prospective studies incorporating rigorous adjustment for key confounders and standardized endpoint assessments are required to determine whether HBV reactivation independently influences survival in patients receiving contemporary therapies for hepatocellular carcinoma.

**Figure 2:**
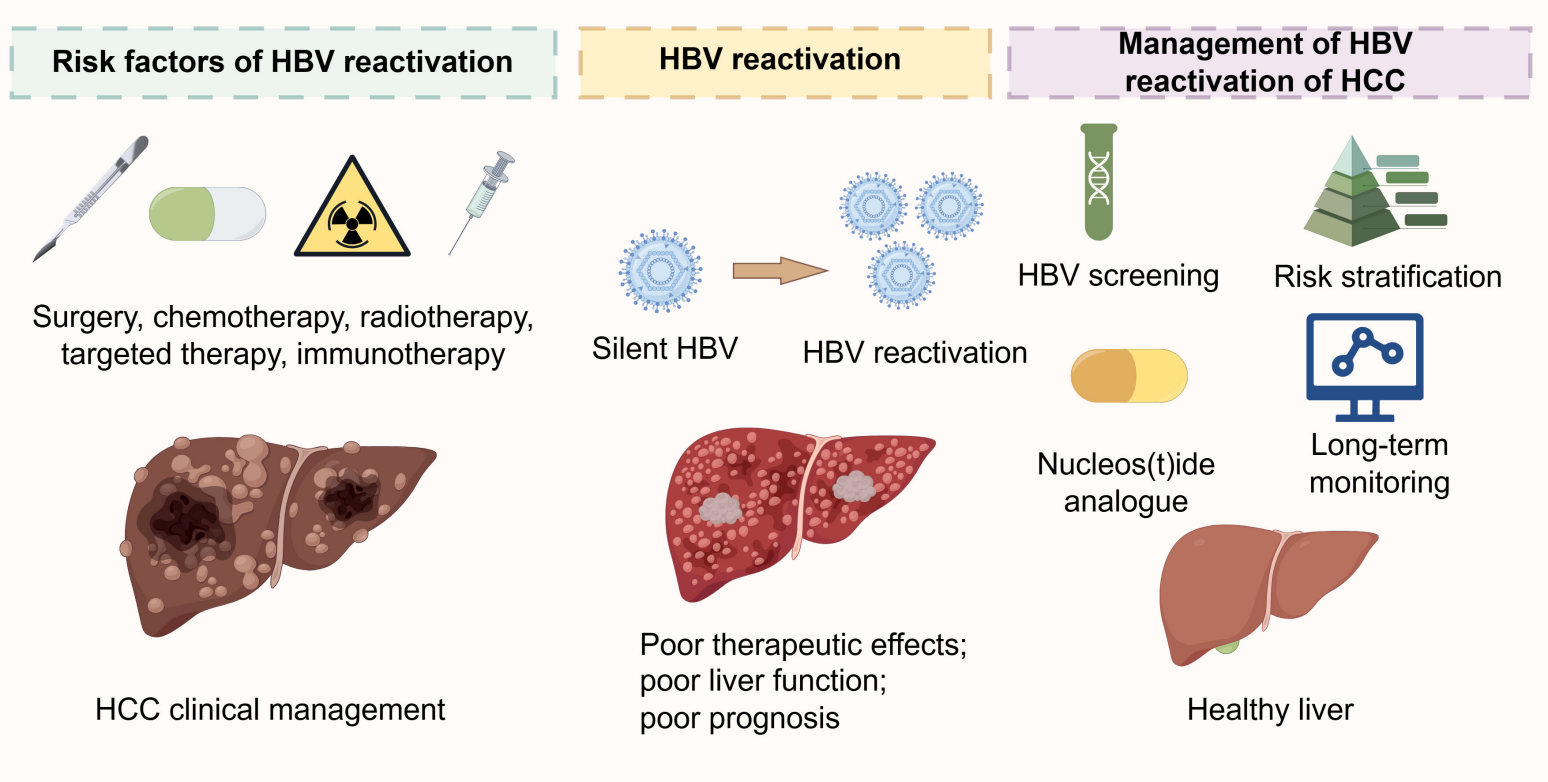
Risk factors and clinical management of HBVr in patients with HCC. The diagram illustrates the key risk factors associated with HBVr during HCC treatment, including surgery, chemotherapy, radiotherapy, targeted therapy, and immunotherapy. It further outlines the impact of HBVr on HCC clinical management, highlighting challenges such as poor therapeutic outcomes and impaired liver function in affected patients, compared to those with a healthy liver or silent HBV infection. HBV, hepatitis B virus; HCC, hepatocellular carcinoma.

The management of HBVr in patients with HCC has advanced into an evidence-based framework built upon risk stratification, prophylactic antiviral therapy, and multidisciplinary coordination (20). However, as treatment paradigms evolve toward increasingly complex regimens, particularly those involving immunotherapies and combination strategies, significant challenges and unmet needs remain. Future advancements will necessitate not only refinements to existing protocols but also innovative approaches that integrate insights from virology, immunology, and global health implementation (**Figure 2**).

A key unresolved challenge is the development of more precise risk prediction tools. Current models depend largely on serological markers and treatment-type classifications; however, growing evidence supports the incorporation of host genetic factors, viral genotypic variations, and quantitative anti-HBc antibody titers (90). This is especially pertinent for HBsAg-negative/anti-HBc-positive individuals, in whom the risk of reverse seroconversion remains difficult to predict under modern immuno-oncologic treatments. Likewise, the continual introduction of novel immunotherapeutic agents and combination regimens underscores the urgency for updated, validated risk stratification models derived from large prospective cohorts. Promising biomarkers such as HBV RNA, pregenomic RNA, and hepatitis B core-related antigen (HBcrAg) may offer more accurate quantification of intrahepatic viral activity and serve as early indicators of reactivation risk, particularly in patients undergoing B-cell-depleting therapies or immune checkpoint inhibition (91).

The long-term objective in HBVr management is to advance beyond viral suppression toward functional or complete cure. Although current NAs effectively inhibit viral replication, they do not eradicate the cccDNA reservoir, which sustains reactivation potential under immunosuppression. Next-generation antiviral approaches aimed at disabling cccDNA, through CRISPR-Cas9 gene editing, transcriptional silencing, or epigenetic modulation, are under active investigation. Additionally, recent studies suggest that co-delivery systems comprising HBx-targeting siRNA and IL-12-encoding plasmids can inhibit HBV replication, reverse virus-mediated immunosuppression, and rejuvenate antiviral immunity, representing a promising therapeutic strategy against HBV infection and hepatocarcinogenesis (92). Success in these innovative drugs could not only prevent HBVr but also redefine the long-term management of HBV-related HCC, potentially obviating the need for prolonged prophylaxis.

Immunotherapy-specific research constitutes another critical frontier. While ICIs have transformed cancer treatment, they engender complex interactions with viral pathogens such as HBV. Current efforts focus on developing HBV-specific cellular therapies, including CAR-T and TCR-T cells, capable of simultaneously targeting tumor and viral antigens, thereby reducing reactivation risk while augmenting antitumor immunity. For instance, SCG101, an autologous T-cell product engineered with a high-affinity TCR against HBV, has demonstrated compelling antiviral and antitumor activities in an early trial involving patients with advanced, treatment-refractory HBV-HCC. A single infusion led to rapid T-cell expansion, marked HBsAg decline, and significant tumor regression, including one complete response sustained for 27 months, with a manageable safety profile dominated by transient cytokine release syndrome (85, 89). Furthermore, the impact of prophylactic NAs on ICIs efficacy remains an urgent clinical question as combination therapies become standard of care. Elucidating the interactions between antiviral prophylaxis, immune activation, and the tumor microenvironment may reveal synergistic mechanisms that improve overall outcomes.

The increasing use of combination therapies for HCC, such as ICIs combined with tyrosine kinase inhibitors, vascular-directed interventions, or cellular infusions, introduces additional complexity into HBVr risk management. These regimens often provoke more profound and multifactorial immunosuppression than monotherapies, necessitating customized monitoring and potentially extended prophylactic measures. Real-world registries and post-marketing surveillance will be essential for documenting reactivation rates in these populations and informing evidence-based guideline updates. From a global health perspective, translating evidence into practice remains particularly challenging in resource-limited regions with high HBV and HCC burdens. Implementing widespread screening and ensuring access to high-barrier NAs are critical steps that demand coordinated efforts among governmental health agencies, non-governmental organizations, and international consortia. Point-of-care diagnostics and low-cost generic antivirals could substantially narrow care disparities and reduce reactivation-related morbidity in underserved communities (38, 93). Finally, the design of future clinical trials must incorporate universal HBV screening and standardized prophylactic protocols. Inconsistent definitions and reporting of HBVr have hindered cross-trial comparisons and meta-analyses. To improve clarity and comparability, HBVr should be explicitly categorized into virological reactivation, defined as a rise in HBV DNA levels above baseline with or without serological changes, and clinical hepatitis flare, defined as an increase in ALT or bilirubin associated with HBV replication. Using these uniform endpoints, including virological reactivation, clinical hepatitis flare, and cancer treatment interruptions due to HBVr, would improve the generalizability of results and facilitate stronger guideline recommendations. Moreover, clinical trials should prioritize the inclusion of diverse populations, especially from HBV-endemic regions, to ensure global applicability of findings (94).

In conclusion, although current strategies have substantially reduced the incidence of HBVr in HCC patients receiving immunosuppressive therapies, the future demands a more sophisticated, innovative, and equitable approach. Progress in biomarker discovery, novel antiviral agents, immunotherapy-tailored solutions, and pragmatic clinical trial design will collectively shape the next generation of HBVr management frameworks (43, 95). Through collaborative science and equitable implementation, we can aspire to eliminate HBVr as an obstacle to effective cancer therapy and improve survival outcomes for patients with HBV-related HCC.
